# Supplementary material for: Two Loci Contribute to Age-Related Hearing Loss Resistance in the Japanese Wild-Derived Inbred MSM/Ms Mice
Source: Biomedicines. 2022 Sep 7;10(9):2221. doi: 10.3390/biomedicines10092221 (PMC9496148; doi:10.3390/biomedicines10092221)
Supplement: Supplementary file 1 [file biomedicines-10-02221-s001.zip › biomedicines-1863754-supplementary.pdf]

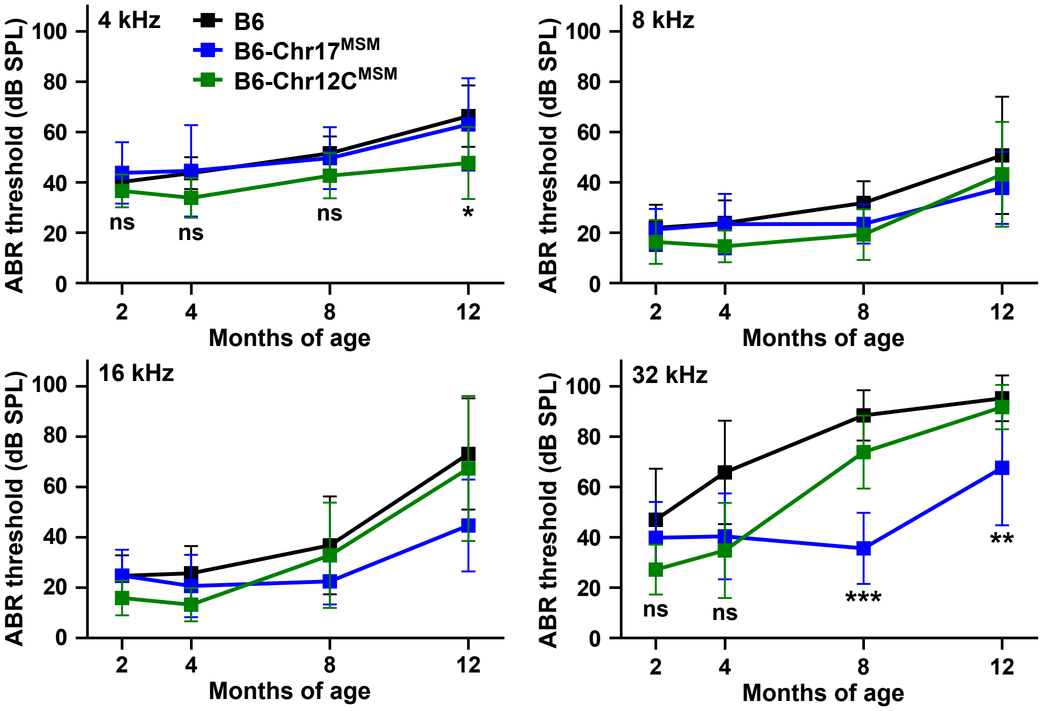

Figure S1. Comparison of the ABR thresholds at 4, 8, 16, and 32 kHz sound stimuli among male mice of the B6, B6-Chr17^{MSM}, and B6-Chr12C^{MSM} strains at 2 (B6, *n* = 15; B6-Chr17^{MSM}, *n* = 16; B6-Chr12C^{MSM}, *n* = 10), 4 (B6, *n* = 15; B6-Chr17^{MSM}, *n* = 15; B6-Chr12C^{MSM}, *n* = 10), 8 (B6, *n* = 14; B6-Chr17^{MSM}, *n* = 16; B6-Chr12C^{MSM}, *n* = 12), and 12 (B6, *n* = 15; B6-Chr17^{MSM}, *n* = 18; B6-Chr12C^{MSM}, n = 11) months of age. The means (squares) and SDs (error bars) of the ABR thresholds are shown. The strain effects by companions of the ABR thresholds between B6-Chr17^{MSM} and B6-Chr12C^{MSM} strains were considered significant at 4 kHz ($F_{Strain\ (1,\ 100)} = 13.88$, $p = 0.0003$) and 32 kHz ($F_{Strain\ (1,\ 100)} = 11.97$, $p = 0.0008$), and not significant at 8 kHz ($F_{Strain\ (1,\ 100)} = 1.82$, $p = 0.1803$) and 16 kHz ($F_{Strain\ (1,\ 100)} = 1.833$, $p = 0.1789$) by two-way ANOVA. The asterisks and "ns" symbols indicate significant (*$p < 0.05$, **$p < 0.01$, and ***$p < 0.001$) and no significant differences, respectively, between the ABR thresholds of the B6-Chr17^{MSM} and B6-Chr12C^{MSM} strains at the same age in each graph, determined using Sidak' s (4 kHz) and Tukey' s (32 kHz) multiple comparison tests after two-way ANOVA.

Table S1. Simple sequence length polymorphism (SSLP) markers used for genotyping of C57BL/6J (B6) and MSM/Ms (MSM) alleles in generations of the congenic lines of *ahl* loci.

Chromosome	SSLP marker	Position (bp, GRCm39)	B6 allele	MSM allele	Forward primer (5'-3')	Reverse primer (5'-3')
12	*D12Mit103*	6,006,981–6,007,112	131-bp	> 131-bp*	ACTATGGTGAAATCATACCCACG	ATCAATGGATCTTTTTTGGTGG
	D12Iga11	9,081,513–9,081,639	123-bp	< 123-bp*	GTAATGTGTGGAGTGAACACAGAG	TAGAGGGAGTTTCATGACAGCTGG
	D12Iga1	11,329,836–11,329,944	108-bp	> 108-bp*	CTTGTTTTGACCTGCTTGGTCG	GTCCTACAGCAACAACTAGG
	D12Mit169	15,341,026–15,341,142	115-bp	137-bp	TTACCCCATATCATATTTGGTCA	TTTTAAAATCACATAGCCTGGTAGC
	D12Iga5	21,232,046–21,232,239	193-bp	> 193-bp*	GCTTCAACTCATTTGAACCTTGAAGG	ACAGCATGTAGCTGGTGCTGG
	D12Mit83	26,285,176–26,285,320	148-bp	157-bp	AGACTGTGGAAAATTAAAACACAGC	GTGCACACATATACATGCAAACA
	D12Mit136	30,223,737–30,223,883	145-bp	156-bp	TTTAATTTTGAGTGGGTTTGGC	TTGCTACATGTACACTGATCTCCA
	D12Mit146	33,238,577–33,238,728	152-bp	141-bp	ACCCCCATTTCTTTACAATTCC	CAAATATGGAAAAAGCATATGTGT
	D12Mit222	38,827,281–38,827,392	113-bp	86-bp	TTTAAAAACAACAACAACAAAAAAGG	ATCTGGGTTTTGAAATAAGAGCC
	D12Iga6	44,426,633–44,426,787	154-bp	< 154-bp*	GGTCAATCCTAAACACATGTGC	CATTATACATACTTCCCTACCCATCCC
	D12Mit64	50,505,009–50,505,146	140-bp	124-bp	CTCCTTGAGATCTGAACACTTGT	GGGCTGGTGGTTTGTCTCT
	D12Mit126	53,075,369–53,075,504	137-bp	153-bp	GGATTATAGGCATGCACTAGGC	TGGGGAGGTGGGTATTAACA
	D12Mit190	59,775,252–59,775,378	125-bp	111-bp	CCCTTGCTATCTTTCAAACCC	TCATAGCAGGTTTATAGGATGTGTG
	D12Mit69	65,510,916–65,511,019	107-bp	98-bp	GAAGAGAGGACATTGCACTGG	AGTTACTGAAGCATAGACCAACCC
	D12Mit34	69,846,626–69,846,796	173-bp	162-bp	GACCACCAGGGCTATTACACA	TGCCAATCTTCACTCATGTACC
17	*D17Mit246*	8,678,516–8,678,617	102-bp	110-bp	GTGGTCTATTTTAAAAAATGAAAAACG	GAGAACCAACTTCAACAAGTTGC
	D17Mit81	31,123,372–31,123,496	122-bp	102-bp	CAATCTATCTCATATGCATCTCTGTG	GTCTGGTGCACCTGTCCTC
	D17Mit176	43,051,185–43,051,352	171-bp	109-bp	GCCTAACTAGACTACAAGCCTTGC	GTAGGTACATACAACCCACATACAGG
	D17Mit274	53,845,536–53,845,735	199-bp	< 199-bp*	ACTCCTTNGGGACCTGCATT	ACCGCTCAGGGAGTGCACTT
	D17Mit20	57,534,447–57,534,623	177-bp	193-bp	AGAACAGGACACCGGACATC	TCATAAGTAGGCACACCAATGC
	D17Mit150	59,260,956–59,261,068	114-bp	128-bp	CTGTTGGATTCATTCATTTAGTGG	TATAGGGACAAACTCCCTGATAGG
	D17Mit4	62,262,073–62,262,170	95-bp	102-bp	GCTGTGCTTCCACACTCAGT	TTTCTGAAAAAGCCTCTCAA
	D17Mit253	63,248,701–63,248,931	230-bp	218-bp	GTGACTGAGGGAGTCATT	CAAGAGAATCACACATATTG
	D17Mit89	63,946,030–63,946,144	113-bp	136-bp	TCAAGTCACAACTGGGACCA	TTGAAATTCTGCCACAGATATCT
	D17Mit91	65,637,246–65,637,357	104-bp	110-bp	GGTTGTTCTCTGGGATCTGC	TTTTCCACTTCAAAAAGGTTAGTG
	D17Mit53	67,480,210–67,480,344	136-bp	126-bp	ATTAGTCCATCACAAATGATTTGG	TTTTGCACAGGAATAGAAACCC
	D17Mit185	68,758,425–68,758,612	188-bp	180-bp	AAAAGGGACAACATACCCAGG	CATCCACTCTGCTTGTATTAGGG
	D17Mit256	69,733,483–69,733,606	124-bp	< 124-bp*	GTGACTGAGGGAGTCATTATGGC**	CAAGAGAATCACACATATTGGAC**
	D17Mit141	73,801,567–73,801,676	111-bp	95-bp	TTGTATTTGTGTATCTGTAAGCATGC	AAATATGACAAGTTGTGTCCTAGCC
	D17Mit74	80,049,879–80,050,031	157-bp	130-bp	CTTCCTTCGTTCCTTCATTCC	CTCTGGAAACTACATGGTGCTG
	D17Mit41	84,643,031–84,643,233	203-bp	222-bp	TGCTTGCTGCTTTCTCAGAA	GATCTGCCTGTCTCCTTAGTGC
	D17Mit123	93,507,047–93,507,179	131-bp	159-bp	CACAAGGAGGGAGCCTGTAG	CACCGTAAGAGTCTAATAATAAGGGG

*The alleles indicate the size differences detected by agarose gel electrophoresis.

**The primer sequences were redesigned.

Table S2. Single nucleotide polymorphism (SNP) markers used for genotyping of B6 and MSM alleles in generations of the congenic lines of *ahl* loci.

Chromosome	SNP ID	Position (bp, GRCm39)	Allele B6	Allele MSM	Forward primer (5'-3')	Reverse primer (5'-3')
12	rs13462802	9,629,170	A	G	CAAAACCTTGCCAGCACCGGTACCC	CACTGTGGGCAGGCCATTCACTGCC
	rs29147141	9,757,239	A	G	TGTGGCGAGAAACCAAGGTAAACAC	GCATCACATGAGCTGAACTAGTATC
	rs240733211	9,904,384	C	T	GTCCATCCCTCTAAACACAATGGAG	ACAAGCACTAGACAGCAAGCCAGAG
	rs36642807	10,049,821	C	T	ACCAAGAAGTGTGATCCCAGAGAAG	TCAGCTGGCATTCACCTGTCACAGG
	NA*	10,132,533	T	G	ATCAAAACATGAGTATGAGGGCTTG	TGTGTGTATATGCTGGGGATCTGAG
	NA	10,444,646	C	T	TGCAGTTTGGAGTGAACCATCTGGG	TCCAAGGAGAAGATTGGTGAGTAGG
17	rs29514721	52,211,146	C	T	AGGATGCACATCAGGGTACACAGAG	CTGTGCATGTGCTACAGAGCGGAAG
	rs29663331	52,813,673	T	C	CCCTGCAGCGCTCCTTGTGGCTTTC	CCTTTTCTCAGGCATCACATAATCC
	rs236845319	53,306,617	C	T	CCCCAGATATCTGTGCCTCCTGGTG	AGAATCAGAAGGAAGAGGTCATAGG
	rs107832179	53,306,620	C	T		
	rs108659421	53,705,721	C	T	CTCAGAGGTGCTAATATGGGTGGTG	GTTAGTAATGTTTGGCAGGCCTGAG
	rs108515592	53,785,930	A	G	GGGTTCCCAACCCGCCGGCTTAGAG	CTCAGTACCACCATTGTTATCTGGG
	rs46197349	53,814,813	G	A	TCCCGGTCTAAGGAATGGTTTTCAC	CAGCAAACAGTGCTACAAAGACTCC
	rs47518120	57,305,460	T	C	TGGTCTGATGCAAGCCAGGTCATCC	GGGATAGCAGGCTCTGGTCAGGACC
	rs264410846	57,305,472	A	C		
	rs108361393	60,304,776	G	A	GGAGTTTCCCTGAAATTTCATCTCC	TGCTAACACTTTATTGATGTACCTC
	rs262014614	60,407,268	A	G	TCTACTGAGGAGCCATATAAAACGG	CTCAAAGGGACTATGAACACTTGGG
	rs220895080	60,512,297	A	G	TTGTTCAAGTGAAATACAGAGGAGG	ACTGGATTAAAGGAACTGAAGAAGG
	rs108786555	60,812,085	C	T	CATAACACAGCAAAGAGCCAATGGG	CTCACAATCCTAGAGCTTCCTTGAG
	rs108804059	60,812,097	C	T		
	rs108073246	61,311,171	A	G	GAGTTGTCCACTCTTTCCTTGTTCG	GTAGGAGGAATGAGAACAATGGAGG
	rs107710832	61,812,175	T	C	TTTCATCCCCATTCCTCCATCACTG	CTTGGTGGCTTACATTTACAGAGGG
	rs108140441	63,277,157	T	C	CATAACTCCTGCGGCAGGGCATCTG	TGTCACAACTGTGCAGGTACCTGGG
	rs231820494	63,310,397	A	C	AATCCTGAGCAAATGGTTTCTAACC	ACATGACACCTTCTAGAAAACACTC
	rs237461892	63,409,058	C	T	GCCTCTCCAGAGATTTCACAGCACC	CGCTACATAAACCCTCTAATCTCCC
	rs108274381	63,509,598	T	C	AAGCCCACACGTCACACATGCCCAC	CAGTCACTGTGTGGATTAGCCTCAC
	rs245957064	63,711,574	C	T	TTTGGGGCATATCTCATTTGGACTG	TATATGAGGACATTCATCCACAGGG
	rs50195359	64,110,812	G	A	CTAGAGGGCGCAGTTGCGTTAACTG	CATTTTGGGGTTTGGGTTCTCCTTG
	rs223120600	64,559,156	G	A	AGCTGTGCTGTGTGCAGCATGGCAG	GACTGCCTGTCTGTCCAGCACAATG
	rs50350231	66,620,337	G	A	CTCTTAGTCAACGAACCATCTCTCC	AGCTTCTACCATAACACCGAAAGCC
	rs46840662	66,643,823	C	T	ATCTGCACGGAGACAGAGTGATGCC	CACTAGGAAAGAGGGACACAGCAGC
	rs238225225	66,757,336	C	A	CTTGCTCCAGGCACTGCTTCTACTC	GGATTTCGAATTCGGGACTGTCATC
	rs33702452	67,110,625	T	C	CAGTCTTCAGGGCTGGGTAGCTGAG	CTGTACTCTAGAGAGTGGGGACAGG
	rs51713859	67,937,864	A	G	CTTGTCCTTGCTGTACCCTCATCCC	TTTTCTCAGAGACAGAGTCAGCCAC
	rs46754501	67,939,859	A	G	CAGTGACAGCTGTCTTGTGCTGCAC	TGATACCCTTGTCTCTAAGGCTTCC
	rs48681006	68,306,726	C	T	CCAGCTGCAGAAGAGAGCCCATGGG	ACAGTTACATCCTGCTTGAGCCAGG
	rs108475928	68,820,252	A	G	TGTCTGGGTGATGTCTATCTGTCTC	CCAGGTGGTGTTTTCTGTGCTCTAC
	rs219141467	69,419,671	G	A	CTTGATGATACTCCTGGGGACTAGG	AAGTCACGTTAGAGGGACACGTAGG
	rs48686235	69,599,668	A	C	CTACCAAGCATCCCCTACTCCTCAG	AAGTCTCTCACATGACTAAGCCAGG
	rs50108016	69,699,066	C	T	TGCCCATTTTTAAGTAGGTCATTGG	ACATCAGGGTGTATGTATACACTTG

*NA, Not annotated.

Table S3. SNPs used for linkage analysis of the loci associated with ARHL resistance of C57BL/6J-Chr 12C^{MSM}/Ms (B6-Chr12C^{MSM}) strain.

SNP ID	Position	Allele		SNP ID	Position	Allele	
	(bp, GRCm39)	B6	MSM		(bp, GRCm39)	B6	MSM
rs50764161	3,337,722	A	G	*rs47371162*	47,260,614	C	T
rs36622082	6,929,135	G	T	*rs31737371*	48,435,755	T	A
rs37903846	11,126,865	C	T	*rs29166012*	50,624,504	T	C
rs50765676	13,137,086	T	C	*rs50212735*	50,881,708	G	A
rs49237917	15,782,050	G	A	*rs52013132*	52,557,694	C	T
rs51940660	31,039,254	T	C	*rs48374396*	52,960,273	T	G
rs50070279	32,478,428	A	G	*rs51589552*	54,632,477	G	A
rs47379643	32,484,388	G	C	*rs31956789*	58,103,451	G	A
rs31644585	34,996,266	G	A	*rs46243707*	62,269,744	C	A
rs36408407	37,640,036	G	A	*rs52560443*	64,532,806	A	G
rs36244327	40,054,917	A	C	*rs32174021*	67,212,252	A	G
rs29187114	42,243,063	A	G	*rs36287012*	68,655,987	G	T
rs37058940	42,439,730	G	T	*rs48876788*	72,152,821	C	T
rs46833947	45,947,086	A	C				

Table S4. Age, frequency, and their interaction effects on the ABR thresholds detected by two-way ANOVA analysis between 1 month of age (1M) and each corrersponding age in male mice of the B6 and MSM strains, and the age effects on the same frequency detected by Sidak's and Tukey's multiple comparisons tests after two-way ANOVA.

Strain	Months of age for comparison	Mean difference ± SD with 1M (dB SPL)	Sourse of variation	1M vs Each corrersponding age			Multiple comparisons test			
				df	*F* value	*p* value	4 kHz	8 kHz	16 kHz	32 kHz
B6	2	4.286±14.91	Age	1, 92	3.408	0.0681	-	-	-	-
			Frequency	3, 92	22.91	< 0.0001***				
			Frequency × Age	3, 92	0.9065	0.4411				
	4	10.57±18.62	Age	1, 92	18.2	< 0.0001***	0.9885	0.892	0.9997	< 0.0001***
			Frequency	3, 92	33.66	< 0.0001***				
			Frequency × Age	3, 92	6.36	0.0006***				
	8	22.98±21.32	Age	1, 88	90.78	< 0.0001***	0.2019	0.0646	0.1113	< 0.0001***
			Frequency	3, 88	49.84	< 0.0001***				
			Frequency × Age	3, 88	15.92	< 0.0001***				
	12	42.16±21.21	Age	1, 92	154.2	< 0.0001***	0.0035**	0.0001***	< 0.0001***	< 0.0001***
			Frequency	3, 92	15	< 0.0001***				
			Frequency × Age	3, 92	4.605	0.0048**				
	16	54.91±15.59	Age	1, 80	448.6	< 0.0001***	< 0.0001***	< 0.0001***	< 0.0001***	< 0.0001***
			Frequency	3, 80	16.92	< 0.0001***				
			Frequency × Age	3, 80	3.427	0.021*				
	20	62.76±11.56	Age	1, 96	1176	< 0.0001***	< 0.0001***	< 0.0001***	< 0.0001***	< 0.0001***
			Frequency	3, 96	22.12	< 0.0001***				
			Frequency × Age	3, 96	1.859	0.1418				
	24	67.23±10.42	Age	1, 72	1339	< 0.0001***	< 0.0001***	< 0.0001***	< 0.0001***	< 0.0001***
			Frequency	3, 72	12.35	< 0.0001***				
			Frequency × Age	3, 72	5.47	0.0019**				
MSM	2	-1.319±10.97	Age	1, 80	0.8176	0.3686	-	-	-	-
			Frequency	3, 80	46.47	< 0.0001***				
			Frequency × Age	3, 80	0.206	0.892				
	4	8.380±12.57	Age	1, 92	18.16	< 0.0001***	0.0938	0.2204	0.1518	0.1027
			Frequency	3, 92	23.68	< 0.0001***				
			Frequency × Age	3, 92	0.03164	0.9924				
	8	6.474±12.05	Age	1, 76	24.19	< 0.0001***	0.0001***	0.7342	0.9965	0.1924
			Frequency	3, 76	78.91	< 0.0001***				
			Frequency × Age	3, 76	3.332	0.0239*				
	12	9.594±13.01	Age	1, 88	55.37	< 0.0001***	< 0.0001***	0.179	0.3612	0.0015**
			Frequency	3, 88	37.47	< 0.0001***				
			Frequency × Age	3, 88	2.326	0.0802				
	16	9.236±11.79	Age	1, 76	39.41	< 0.0001***	0.0004***	0.1129	0.2828	0.0001***
			Frequency	3, 76	55.76	< 0.0001***				
			Frequency × Age	3, 76	1.793	0.1557				
	20	12.25±13.08	Age	1, 72	34.25	< 0.0001***	0.001***	0.0071**	0.2004	0.039*
			Frequency	3, 72	26.13	< 0.0001***				
			Frequency × Age	3, 72	0.6618	0.5782				
	24	11.77±14.04	Age	1, 72	24.61	< 0.0001***	0.0119*	0.0426*	0.5611	0.0198*
			Frequency	3, 72	20.88	< 0.0001***				
			Frequency × Age	3, 72	0.6183	0.6054				

*$p < 0.05$, **$p < 0.01$, and ***$p < 0.001$.

Table S5. Age, frequency, and their interaction effects on the DPOAE amplitudes detected by two-way ANOVA analysis between 1 month of age (1M) and each corresponding age in male mice of the B6 and MSM strains, and the age effects on the same frequency detected by Sidak's and Tukey's multiple comparisons tests after two-way ANOVA.

Strain	Months of age	Mean difference ± SD	Sourse of	1M vs each corresponding age			Multiple comparisons test				
	for comparison	with 1M (dB SPL)	variation	df	*F* value	*p* value	8 kHz	11.3 kHz	16 kHz	22.6 kHz	32 kHz
B6	2	-5.071±11.34	Age	1, 100	14.39	0.0003***	0.0175*	0.0997	0.6052	0.9997	0.512
			Frequency	4, 100	45.55	< 0.0001***					
			Frequency × Age	4, 100	1.081	0.3701					
	4	-1.922±13.53	Age	1, 115	1.298	0.257	-	-	-	-	-
			Frequency	4, 115	30.51	< 0.0001***					
			Frequency × Age	4, 115	5.048	0.0009***					
	8	14.12±15.71	Age	1, 110	43.1	< 0.0001***	0.9641	0.9635	0.2797	< 0.0001***	< 0.0001***
			Frequency	4, 110	14.98	< 0.0001***					
			Frequency × Age	4, 110	10.12	< 0.0001***					
	12	24.26±13.13	Age	1, 115	252.7	< 0.0001***	> 0.9999	0.0131*	< 0.0001***	< 0.0001***	< 0.0001***
			Frequency	4, 115	18.79	< 0.0001***					
			Frequency × Age	4, 115	25.96	< 0.0001***					
	16	32.97±9.682	Age	1, 90	588	< 0.0001***	0.001***	< 0.0001***	< 0.0001***	< 0.0001***	< 0.0001***
			Frequency	4, 90	12.62	< 0.0001***					
			Frequency × Age	4, 90	14.58	< 0.0001***					
MSM	2	1.397±13.77	Age	1, 120	1.097	0.2971	-	-	-	-	-
			Frequency	4, 120	75.84	< 0.0001***					
			Frequency × Age	4, 120	0.08556	0.9868					
	4	1.315±13.61	Age	1, 120	1.007	0.3176	-	-	-	-	-
			Frequency	4, 120	76.68	< 0.0001***					
			Frequency × Age	4, 120	0.8416	0.5014					
	8	-1.614±13.08	Age	1, 105	3.272	0.0733	-	-	-	-	-
			Frequency	4, 105	183.5	< 0.0001***					
			Frequency × Age	4, 105	1.073	0.3739					
	12	-1.168±13.07	Age	1, 105	1.101	0.2965	-	-	-	-	-
			Frequency	4, 105	108.2	< 0.0001***					
			Frequency × Age	4, 105	0.9546	0.4357					
	16	1.566±13.97	Age	1, 95	1.4	0.2397	-	-	-	-	-
			Frequency	4, 95	85.03	< 0.0001***					
			Frequency × Age	4, 95	0.779	0.5416					
	20	-0.2409±13.14	Age	1, 90	0.04386	0.8346	-	-	-	-	-
			Frequency	4, 90	105.1	< 0.0001***					
			Frequency × Age	4, 90	0.4716	0.7564					
	24	3.210±12.73	Age	1, 95	7.42	0.0077**	0.9812	0.9994	0.2013	0.4459	0.1655
			Frequency	4, 95	89.2	< 0.0001***					
			Frequency × Age	4, 95	1.082	0.37					

*$p < 0.05$, **$p < 0.01$, and ***$p < 0.001$.

Table S6. Age, area, and their interaction effects on the IHC and OHC survival rates detected by two-way ANOVA analysis between 2 month of age (2M) and each corresponding age in male B6 and MSM mice, and the age effects on the same area detected by Sidak's and Tukey's multiple comparisons tests after two-way ANOVA.

Strain	Cell type	Months of age for comparison	Mean difference ± SD with 2M (%)	Sourse of variation	2M vs each corresponding age			Multi comparison test			
					df	*F* value	*p* value	4 kHz	8 kHz	16 kHz	32 kHz
B6	IHC	10	21.36±14.45	Age	1, 40	35.14	< 0.0001***	0.9894	0.5638	0.002**	0.0006***
				Area	3, 40	3.617	0.0211*				
				Area × Age	3, 40	3.609	0.0213*				
		18	20.18±14.78	Age	1, 40	38.71	< 0.0001***	> 0.9999	0.4312	0.0009***	< 0.0001***
				Area	3, 40	6.593	0.001**				
				Area × Age	3, 40	6.582	0.001**				
		24	36.96±11.96	Age	1, 36	145.5	< 0.0001***	0.0445*	< 0.0001***	< 0.0001***	< 0.0001***
				Area	3, 36	4.126	0.013*				
				Area × Age	3, 36	4.118	0.0131*				
	OHC	10	13.74±10.28	Age	1, 40	20.39	< 0.0001***	0.0106*	0.3875	0.4036	0.0468*
				Area	3, 40	0.6313	0.5992				
				Area × Age	3, 40	0.6365	0.5959				
		18	50.80±21.29	Age	1, 40	224.4	< 0.0001***	< 0.0001***	< 0.0001***	0.1479	< 0.0001***
				Area	3, 40	18.51	< 0.0001***				
				Area × Age	3, 40	18.52	< 0.0001***				
		24	92.57±7.353	Age	1, 36	2710	< 0.0001***	< 0.0001***	< 0.0001***	< 0.0001***	< 0.0001***
				Area	3, 36	5.419	0.0035**				
				Area × Age	3, 36	5.429	0.0035**				
MSM	IHC	10	5.709±5.389	Age	1, 40	11.99	0.0013**	0.3134	0.1756	0.6838	0.2052
				Area	3, 40	0.1589	0.9233				
				Area × Age	3, 40	0.1587	0.9235				
		18	1.596±8.249	Age	1, 40	0.4026	0.5293	-	-	-	-
				Area	3, 40	0.2045	0.8927				
				Area × Age	3, 40	0.2003	0.8955				
		24	2.666±6.622	Age	1, 40	1.709	0.1986	-	-	-	-
				Area	3, 40	0.07185	0.9747				
				Area × Age	3, 40	0.06881	0.9762				
	OHC	10	14.13±10.09	Age	1, 40	47.59	< 0.0001***	< 0.0001***	< 0.0001***	0.9179	0.9989
				Area	3, 40	8.832	0.0001***				
				Area × Age	3, 40	8.83	0.0001***				
		18	23.52±14.76	Age	1, 40	86.09	< 0.0001***	< 0.0001***	< 0.0001***	0.5031	0.9871
				Area	3, 40	15	< 0.0001***				
				Area × Age	3, 40	15	< 0.0001***				
		24	51.36±22.89	Age	1, 40	385.1	< 0.0001***	< 0.0001***	< 0.0001***	< 0.0001***	0.9192
				Area	3, 40	42.23	< 0.0001***				
				Area × Age	3, 40	42.22	< 0.0001***				

*$p < 0.05$, **$p < 0.01$, and ***$p < 0.001$.

Table S7. Strain, age, and their interaction effects on the ABR thresholds at 4, 8, 16, and 32 kHz detected by two-way ANOVA analysis between B6 and C57BL/6J-Chr 17^{MSM}/Ms (B6-Chr17^{MSM}) strains, and between B6-*Cdh23* $^{+/+}$ and B6-Chr17^{MSM} strains, in male mice at 2, 4, 8, 12, and 16 months of age.

Frequency	Sourse of	B6 vs B6-Chr17^{MSM}			B6-*Cdh23* $^{+/+}$ vs B6-Chr17^{MSM}		
(kHz)	variation	df	*F* value	*p* value	df	*F* value	*p* value
4	Strain	1, 138	0.03443	0.8531	1, 125	21.2	< 0.0001***
	Age	4, 138	35.21	< 0.0001***	4, 125	22.3	< 0.0001***
	Age × Strain	4, 138	0.2686	0.8978	4, 125	0.7955	0.5302
8	Strain	1, 138	12.69	0.0005***	1, 125	3.544	0.0621
	Age	4, 138	37.33	< 0.0001***	4, 125	33.14	< 0.0001***
	Age × Strain	4, 138	2.153	0.0775	4, 125	0.4286	0.7878
16	Strain	1, 138	34.7	< 0.0001***	1, 125	7.226	0.0082**
	Age	4, 138	66.1	< 0.0001***	4, 125	32.5	< 0.0001***
	Age × Strain	4, 138	5.116	0.0007***	4, 125	0.9399	0.4433
32	Strain	1, 138	87.98	< 0.0001***	1, 125	26.59	< 0.0001***
	Age	4, 138	45.73	< 0.0001***	4, 125	28.03	< 0.0001***
	Age × Strain	4, 138	9.484	< 0.0001***	4, 125	3.483	0.0098**

**$p < 0.01$ and ***$p < 0.001$.

Table S8. Strain, frequency, and their interaction effects on the DPOAE amplitudes against the f_2 frequencies at 8, 11.3, 16, 22.6, and 32 kHz detected by two-way ANOVA analysis between B6 and B6-Chr17^{MSM} strains, and between B6-*Cdh23*$^{+/+}$ and B6-Chr17^{MSM} strains, in male mice at 4, 8, and 12 months of age.

Months	Sourse of	B6 vs B6-Chr17^{MSM}			B6-*Cdh23*$^{+/+}$ vs B6-Chr17^{MSM}		
of age	variation	df	*F* value	*p* value	df	*F* value	*p* value
4	Strain	1, 115	0.02131	0.8842	1, 105	11.63	0.0009***
	Frequency	4, 115	34.39	< 0.0001***	4, 105	60.19	< 0.0001***
	Frequency × Strain	4, 115	4.896	0.0011**	4, 105	1.167	0.3295
8	Strain	1, 115	40.74	< 0.0001***	1, 110	6.128	0.0148*
	Frequency	4, 115	12.56	< 0.0001***	4, 110	17.98	< 0.0001***
	Frequency × Strain	4, 115	6.946	< 0.0001***	4, 110	0.5769	0.68
12	Strain	1, 125	36.71	< 0.0001***	1, 110	24.83	< 0.0001***
	Frequency	4, 125	8.199	< 0.0001***	4, 110	10.27	< 0.0001***
	Frequency × Strain	4, 125	12.89	< 0.0001***	4, 110	0.5569	0.6944

*p < 0.05, **p < 0.01, and ***p < 0.001.

Table S9. Strain, age, and their interaction effects on the ABR thresholds at 4, 8, 16, and 32 kHz detected by two-way ANOVA analysis between B6 and each *ahl3* congenic strain B6.MSM-*ahl3* /1Tmims (B6.MSM-*ahl3* /1) and B6.MSM-*ahl3* /2Tmims (B6.MSM-*ahl3* /2), and between B6-Chr17^{MSM} and each *ahl3* congenic strain, in male mice at 2, 4, 8, 12, and 16 months of age.

Frequency	Sourse of	B6 vs B6.MSM-*ahl3* /1			B6 vs B6.MSM-*ahl3* /2			B6-Chr17^{MSM} vs B6.MSM-*ahl3* /1			B6-Chr17^{MSM} vs B6.MSM-*ahl3* /2		
(kHz)	variation	df	*F* value	*p* value	df	*F* value	*p* value	df	*F* value	*p* value	df	*F* value	*p* value
4	Strain	1, 119	6.553	0.0117*	1, 125	0.09831	0.7544	1, 125	6.998	0.0092**	1, 131	0.233	0.6301
	Age	4, 119	38.12	< 0.0001***	4, 125	44.49	< 0.0001***	4, 125	31.85	< 0.0001***	4, 131	35.93	< 0.0001***
	Age × Strain	4, 119	0.6336	0.6395	4, 125	0.5535	0.6968	4, 125	0.9372	0.4448	4, 131	1.246	0.2949
8	Strain	1, 119	3.799	0.0536	1, 125	1.391	0.2405	1, 125	28.89	< 0.0001***	1, 131	4.504	0.0357*
	Age	4, 119	42.61	< 0.0001***	4, 125	35.9	< 0.0001***	4, 125	37.2	< 0.0001***	4, 131	29.87	< 0.0001***
	Age × Strain	4, 119	0.4851	0.7467	4, 125	0.3039	0.8749	4, 125	4.198	0.0032**	4, 131	1.237	0.2984
16	Strain	1, 119	2.716	0.102	1, 125	11.83	0.0008***	1, 125	46.83	< 0.0001***	1, 131	2.135	0.1464
	Age	4, 119	64.5	< 0.0001***	4, 125	45.21	< 0.0001***	4, 125	48.24	< 0.0001***	4, 131	30.5	< 0.0001***
	Age × Strain	4, 119	0.2771	0.8923	4, 125	2.527	0.044*	4, 125	4.589	0.0017**	4, 131	0.4963	0.7385
32	Strain	1, 119	0.02016	0.8873	1, 125	33.37	< 0.0001***	1, 125	59.58	< 0.0001***	1, 131	5.788	0.0175*
	Age	4, 119	46.34	< 0.0001***	4, 125	47.33	< 0.0001***	4, 125	36.34	< 0.0001***	4, 131	42.13	< 0.0001***
	Age × Strain	4, 119	0.199	0.9384	4, 125	2.177	0.0754	4, 125	6.002	0.0002***	4, 131	3.664	0.0073**

*$p < 0.05$, **$p < 0.01$, and ***$p < 0.001$.

Table S10. Strain, frequency, and their interaction effects on the DPOAE amplitudes against the f_2 frequencies at 8, 11.3, 16, 22.6, and 32 kHz detected by two-way ANOVA analysis between B6 and B6.MSM-*ahl3* strains, and between B6-Chr17^{MSM} and B6.MSM-*ahl3* /2 strains, in male mice at 4, 8, and 12 months of age.

Months of age	Sourse of variation	B6 vs B6.MSM-*ahl3* /2			B6-Chr17^{MSM} vs B6.MSM-*ahl3* /2		
		df	*F* value	*p* value	df	*F* value	*p* value
4	Strain	1, 115	12.36	0.0006***	1, 90	15.0	0.0002***
	Frequency	4, 115	29.34	< 0.0001***	4, 90	50.41	< 0.0001***
	Strain × Frequency	4, 115	4.082	0.0039**	4, 90	0.4104	0.8008
8	Strain	1, 110	13.59	0.0004***	1, 95	6.299	0.0138*
	Frequency	4, 110	11.79	< 0.0001***	4, 95	14.57	< 0.0001***
	Strain × Frequency	4, 110	5.302	0.0006***	4, 95	0.07799	0.9888
12	Strain	1, 125	2.65	0.1061	1, 110	12.81	0.0005***
	Frequency	4, 125	14.93	< 0.0001***	4, 110	9.497	< 0.0001***
	Strain × Frequency	4, 125	6.918	< 0.0001***	4, 110	0.99	0.4166

*$p < 0.05$, **$p < 0.01$, and ***$p < 0.001$.

Table S11. Strain, age, and their interaction effects on the ABR thresholds at 4, 8, 16, and 32 kHz detected by two-way ANOVA analysis between B6 and B6-Chr12C^{MSM} strains in male mice at 2, 4, 8, and 12 months of age.

Frequency (kHz)	Sourse of variation	B6 vs B6-Chr12C^{MSM}		
		df	*F* value	*p* value
4	Strain	1, 94	15.85	0.0001***
	Age	3, 94	11.63	< 0.0001***
	Age × Strain	3, 94	1.489	0.2225
8	Strain	1, 94	10.21	0.0019**
	Age	3, 94	23.4	< 0.0001***
	Age × Strain	3, 94	0.2939	0.8297
16	Strain	1, 94	4.958	0.0284*
	Age	3, 94	46.63	< 0.0001***
	Age × Strain	3, 94	0.2887	0.8335
32	Strain	1, 94	32.41	< 0.0001***
	Age	3, 94	75.11	< 0.0001***
	Age × Strain	3, 94	3.548	0.0174*

*p < 0.05, **p < 0.01, and ***p < 0.001.

Table S12. Strain, age, and their interaction effects on the ABR thresholds at 4, 8, 16, and 32 kHz detected by two-way ANOVA analysis between B6 and B6.MSM-*ahl10*/Tmims (B6.MSM-*ahl10*) strains, and between B6-Chr12C^{MSM} and B6.MSM-*ahl10* strains, in male mice at 2, 4, 8, and 12 months of age.

Frequency	Sourse of	B6 vs B6.MSM-*ahl10*			B6-Chr12C^{MSM} vs B6.MSM-*ahl10*		
(kHz)	variation	df	*F* value	*p* value	df	*F* value	*p* value
4	Strain	1, 97	16.31	0.0001***	1, 81	0.00343	0.9534
	Age	3, 97	17.08	< 0.0001***	3, 81	12.68	< 0.0001***
	Age × Strain	3, 97	0.387	0.7626	3, 81	1.608	0.1939
8	Strain	1, 97	13.33	0.0004***	1, 81	0.09183	0.7626
	Age	3, 97	23.69	< 0.0001***	3, 81	23.45	< 0.0001***
	Age × Strain	3, 97	0.2044	0.8931	3, 81	0.2525	0.8593
16	Strain	1, 97	10.55	0.0016**	1, 81	0.3543	0.5533
	Age	3, 97	51.07	< 0.0001***	3, 81	41.17	< 0.0001***
	Age × Strain	3, 97	0.1814	0.9088	3, 81	0.2515	0.86
32	Strain	1, 97	37.45	< 0.0001***	1, 81	1.287	0.26
	Age	3, 97	54.79	< 0.0001***	3, 81	71.86	< 0.0001***
	Age × Strain	3, 97	3.329	0.0228*	3, 81	0.2425	0.8664

*$p < 0.05$, **$p < 0.01$, and ***$p < 0.001$.

Table S13. Strain, frequency, and their interaction effects on the DPOAE amplitudes against the f_2 frequencies at 8, 11.3, 16, 22.6, and 32 kHz detected by two-way ANOVA analysis between B6 and B6. MSM-*ahl10* strains in male mice at 4 and 8 months of age.

Months of age	Sourse of variation	B6 vs B6.MSM-*ahl10*		
		df	*F* value	*p* value
4	Strain	1, 140	22.18	< 0.0001***
	Frequency	4, 140	38.76	< 0.0001***
	Frequency × Strain	4, 140	5.562	0.0003***
8	Strain	1, 115	12.18	0.0007***
	Frequency	4, 115	21.56	< 0.0001***
	Frequency × Strain	4, 115	0.7733	0.5448

***$p < 0.001$.

Table S14. Predicted neutral amino acid substitutions between B6 and MSM mice in the protein-coding genes within MSM-*ahl3*/2 and MSM-*ahl10* congenic regions.

Congenic region	Protein	Amino acid substitution			Possible impact		
		Position	B6	MSM	SIFT	PROVEAN	PolyPhen-2
ahl3/2	FBXL17	72	Ala	Thr	Tolerated	Neutral	Benign
	A930002H24RIK	6	Leu	Phe	NA*	Neutral	NA
		15	Thr	Ile	NA	Neutral	NA
		16	Ala	Val	NA	Neutral	NA
		205	Cys	Arg	NA	Neutral	NA
		233	Arg	Gln	NA	Neutral	NA
		242	Glu	Gly	NA	Neutral	NA
		271	Ser	Trp	NA	Neutral	NA
		341	Gly	Glu	NA	Neutral	NA
	PJA2	108	Ala	Thr	Tolerated	Neutral	Benign
		141	Arg	His	Tolerated	Neutral	Benign
		152	Asn	Ser	Tolerated	Neutral	Benign
		291	His	Arg	Tolerated	Neutral	Benign
	MAN2A1	764	Pro	Ser	Tolerated	Neutral	Benign
		924	Ala	Val	Tolerated	Neutral	Benign
	4930583I09RIK	40	Asn	Lys	NA	Neutral	NA
		92	Leu	Trp	NA	Neutral	NA
	TMEM232	217	Val	Met	NA	Neutral	Benign
		237	Phe	Leu	NA	Neutral	Benign
		263	Val	Ile	NA	Neutral	Benign
		360	Val	Ile	NA	Neutral	Benign
		610	Glu	Ala	NA	Neutral	Benign
	TXNDC2	100	Pro	Ser	Tolerated	Neutral	Benign
		103	Gly	Glu	Tolerated	Neutral	Benign
		175	Pro	Ser	Tolerated	Neutral	Benign
		205	Pro	Ser	Tolerated	Neutral	Benign
		219	Gln	Pro	Tolerated	Neutral	Benign
	PPP4R1	4	Asn	Ser	Tolerated	Neutral	Benign
		26	Ser	Pro	Tolerated	Neutral	Benign
		180	Asp	Glu	Tolerated	Neutral	Benign
		236	Ala	Val	Tolerated	Neutral	Benign
	RALBP1	9	Ser	Thr	Tolerated	Neutral	Benign
		53	Val	Ile	Tolerated	Neutral	Benign
		65	Asp	Glu	Tolerated	Neutral	Benign
		183	Ala	Ser	Tolerated	Neutral	Benign
		195	Val	Ala	Tolerated	Neutral	Benign
		617	Ile	Thr	Tolerated	Neutral	Benign
	TWSG1	19	Leu	Phe	Tolerated	Neutral	Benign
	ANKRD12	509	Tyr	Phe	Tolerated	Neutral	Benign
		988	Ala	Val	Tolerated	Neutral	Benign
		1153	Asn	Ser	Tolerated	Neutral	Benign
		1420	Ile	Thr	Tolerated	Neutral	Benign
	DDX11	93	Thr	Ile	Tolerated	Neutral	Benign

*NA, Not annotated.

Table S14. Continued.

Congenic region	Protein	Amino acid substitution			Possible impact		
		Position	B6	MSM	SIFT	PROVEAN	PolyPhen-2
ahl3 /2	MTCL1	90	Gly	Ser	Tolerated	Neutral	Benign
		229	Ala	Thr	Tolerated	Neutral	Benign
		779	Gly	Glu	Tolerated	Neutral	Benign
		780	Ser	Arg	Tolerated	Neutral	Benign
		783	Ser	Pro	Tolerated	Neutral	Benign
	THEMIS3	149	Lys	Glu	Tolerated	Neutral	NA
		228	Thr	Met	Tolerated	Neutral	NA
		313	Ser	Thr	Tolerated	Neutral	NA
		355	Glu	Gln	Tolerated	Neutral	NA
		362	Gln	Arg	Tolerated	Neutral	NA
		562	Val	Ala	Tolerated	Neutral	NA
	PTPRM	607	Met	Val	Tolerated	Neutral	Benign
	LRRC30	120	Val	Ile	Tolerated	Neutral	Benign
	LAMA1	405	Asn	Asp	Tolerated	Neutral	Benign
		822	Val	Ile	Tolerated	Neutral	Benign
		920	Ile	Val	Tolerated	Neutral	Benign
		1295	Arg	Gln	Tolerated	Neutral	Benign
		1376	Ala	Ser	Tolerated	Neutral	Benign
		1646	Arg	Cys	Tolerated	Neutral	Benign
		1800	Gln	Arg	Tolerated	Neutral	Benign
		1839	Thr	Ala	Tolerated	Neutral	Benign
		1853	Arg	Asn	Tolerated	Neutral	Benign
		1925	Met	Thr	Tolerated	Neutral	Benign
		1964	Ser	Gly	Tolerated	Neutral	Benign
		1974	Met	Leu	Tolerated	Neutral	Benign
		2022	Ala	Val	Tolerated	Neutral	Benign
		2522	Ala	Thr	Tolerated	Neutral	Benign
		2872	Ile	Met	Tolerated	Neutral	Benign
		2983	Gly	Ser	Tolerated	Neutral	Benign
		3005	Ser	Ala	Tolerated	Neutral	Benign
	ARHGAP28	223	Ser	Asn	Tolerated	Neutral	Benign
		300	Val	Met	Tolerated	Neutral	Benign
	L3MBTL4	37	Thr	Ala	Tolerated	Neutral	Benign
		66	Ala	Val	Tolerated	Neutral	Benign
	TMEM200C	262	Val	Met	Tolerated	Neutral	Benign
		360	Thr	Asn	Tolerated	Neutral	Benign
		451	Ala	Val	Tolerated	Neutral	Benign
		458	Pro	Ser	Tolerated	Neutral	Benign
	EPB41L3	52	Ala	Thr	Tolerated	Neutral	Benign
		775	Thr	Ala	Tolerated	Neutral	Benign
ahl10	KIF3C	201	Ala	Thr	Tolerated	Neutral	Benign
	ASXL2	351	Ser	Pro	Tolerated	Neutral	Benign
		527	Asp	Glu	Tolerated	Neutral	Benign
		552	Val	Ala	Tolerated	Neutral	Benign
		977	Arg	Lys	Tolerated	Neutral	Benign
		1197	Pro	Ser	Tolerated	Neutral	Benign

Table S14. Continued.

Congenic region	Protein	Amino acid substitution			Possible impact		
		Position	B6	MSM	SIFT	PROVEAN	PolyPhen-2
ahl10	DNMT3A	151	Gln	Pro	Tolerated	Neutral	Benign
	DNAJC27	3	Thr	Ser	Tolerated	Neutral	Benign
	ADCY3	512	Val	Met	Tolerated	Neutral	Benign
	CENPO	30	Ile	Val	Tolerated	Neutral	Benign
		39	Ala	Thr	Tolerated	Neutral	Benign
	NCOA1	863	Thr	Ala	Tolerated	Neutral	Benign
	ITSN2	995	Val	Ile	Tolerated	Neutral	NA
		996	Arg	Thr	Tolerated	Neutral	NA
	FAM228A	32	Leu	Ile	Tolerated	Neutral	Benign
		161	Leu	Pro	Tolerated	Neutral	Benign
		165	Pro	Thr	Tolerated	Neutral	Benign
	WDCP	33	Arg	His	Tolerated	Neutral	Benign
		83	Lys	Met	Tolerated	Neutral	Benign
		243	Gly	Ser	Tolerated	Neutral	Benign
		503	Val	Ile	Tolerated	Neutral	Benign
		543	Lys	Asn	Tolerated	Neutral	Benign
		572	Thr	Met	Tolerated	Neutral	Benign
		581	Val	Ile	Tolerated	Neutral	Benign
	MFSD2B	170	Ala	Thr	Tolerated	Neutral	Benign
	UBXN2A	15	Ala	Val	Tolerated	Neutral	Benign
	ATAD2B	1115	Arg	Gln	Tolerated	Neutral	Benign
	KLHL29	195	His	Arg	NA	Neutral	NA
		978	Val	Met	Tolerated	Neutral	Benign
	APOB	124	Thr	Met	Tolerated	Neutral	Benign
		244	Val	Ile	Tolerated	Neutral	Benign
		1391	Lys	Arg	Tolerated	Neutral	Benign
	GDF7	57	Ala	Thr	Tolerated	Neutral	NA
		244	Asp	Glu	Tolerated	Neutral	NA
	HS1BP3	359	Pro	Thr	NA	Neutral	Benign
	SLC7A15	431	Ala	Val	Tolerated	Neutral	Benign
	LAPTM4A	25	Gly	Ser	Tolerated	Neutral	NA
	MATN3	25	Ser	Pro	Tolerated	Neutral	Benign
		41	Gly	Val	Tolerated	Neutral	Benign
		264	Met	Val	Tolerated	Neutral	Benign
		281	Lys	Arg	Tolerated	Neutral	Benign
		386	Arg	Gln	Tolerated	Neutral	Benign
		390	Ala	Ser	Tolerated	Neutral	Benign
	TTC32	3	Leu	Ala	Tolerated	Neutral	Benign
		70	Asp	Glu	Tolerated	Neutral	Benign
